# Microevolution of *Renibacterium salmoninarum*: evidence for intercontinental dissemination associated with fish movements

**DOI:** 10.1038/ismej.2013.186

**Published:** 2013-10-31

**Authors:** Ola Brynildsrud, Edward J Feil, Jon Bohlin, Santiago Castillo-Ramirez, Duncan Colquhoun, Una McCarthy, Iveta M Matejusova, Linda D Rhodes, Gregory D Wiens, David W Verner-Jeffreys

**Affiliations:** 1 EpiCentre, Department of Food Safety and Infection Biology, Norwegian School of Veterinary Science, Oslo, Norway; 2 Department of Biology and Biochemistry, University of Bath, Bath, UK; 3 Section for Bacteriology, Department of Laboratory Services, Norwegian Veterinary Institute, Oslo, Norway; 4 Marine Scotland Science, Aberdeen, Scotland, UK; 5 NOAA, Northwest Fisheries Science Center, Seattle, WA, USA; 6 USDA, National Centre for Cool and Coldwater Aquaculture, Leetown, WV, USA; 7 Cefas Weymouth Laboratory, The Nothe, Weymouth, UK

**Keywords:** fish pathogens, microbial evolution, next-generation sequencing, *Renibacterium salmoninarum*, transmission history

## Abstract

*Renibacterium salmoninarum* is the causative agent of bacterial kidney disease, a major pathogen of salmonid fish species worldwide. Very low levels of intra-species genetic diversity have hampered efforts to understand the transmission dynamics and recent evolutionary history of this Gram-positive bacterium. We exploited recent advances in the next-generation sequencing technology to generate genome-wide single-nucleotide polymorphism (SNP) data from 68 diverse *R. salmoninarum* isolates representing broad geographical and temporal ranges and different host species. Phylogenetic analysis robustly delineated two lineages (lineage 1 and lineage 2); futhermore, dating analysis estimated that the time to the most recent ancestor of all the isolates is 1239 years ago (95% credible interval (CI) 444–2720 years ago). Our data reveal the intercontinental spread of lineage 1 over the last century, concurrent with anthropogenic movement of live fish, feed and ova for aquaculture purposes and stocking of recreational fisheries, whilst lineage 2 appears to have been endemic in wild Eastern Atlantic salmonid stocks before commercial activity. The high resolution of the SNP-based analyses allowed us to separate closely related isolates linked to neighboring fish farms, indicating that they formed part of single outbreaks. We were able to demonstrate that the main lineage 1 subgroup of *R. salmoninarum* isolated from Norway and the UK likely represent an introduction to these areas ∼40 years ago. This study demonstrates the promise of this technology for analysis of micro and medium scale evolutionary relationships in veterinary and environmental microorganisms, as well as human pathogens.

## Introduction

The causative agent of bacterial kidney disease (BKD) in salmonids, *Renibacterium salmoninarum,* is a Gram-positive slow-growing facultative intracellular pathogen. BKD, a chronic, progressive granulomatous infection, is a major threat to both farmed and wild salmonid fish species worldwide (Fryer and Sanders, 1981; Evelyn, 1993; Evenden *et al.*, 1993; Fryer and Lannan, 1993; Wiens, 2011). It was first reported in the wild Atlantic salmon (*Salmo salar* L.) from rivers in Scotland and in brook and brown trout from the East coast of US in the 1930s (Earp *et al.*, 1953; Smith, 1964).

The genome of *R. salmoninarum* consists of a single circular 3.15-Mbp chromosome with no known plasmids or phage elements (Wiens *et al.*, 2008). As with other specialized intracellular pathogens, there is evidence of genome reduction (Wiens *et al.*, 2008), and it has evolved mechanisms to evade detection by the host immune system (Grayson *et al.*, 2002). *R. salmoninarum* survives, and possibly also replicates, within the macrophages of the kidney (Young and Chapman, 1978; Gutenberger *et al.*, 1997). The bacterium is able to spread horizontally between fish hosts as well as vertically via the ova (Evelyn *et al.*, 1986). Overt symptoms may not be seen until several months post infection, thus providing ample opportunity for horizontal transmission through stocks. Transovarial transmission additionally provides a mechanism for global dissemination of *R. salmoninarum* via commercial activity. These factors, coupled with a lack of efficient therapy and vaccine regimens for this disease (Elliott *et al.*, 1989), have, in some countries, resulted in the imposition of movement controls on premises confirmed as positive for BKD, or the destruction of infected animals, disinfection and fallowing of premises (Richards, 2011).

There is continued speculation relating to the likely origins and spread of BKD. It is frequently detected in wild and hatchery-bred Pacific salmon (*Oncorhynchus* spp.) in both freshwater and oceanic phases (Banner *et al.*, 1986; Kent *et al.*, 1998; Meyers *et al.*, 2003; Arkoosh *et al.*, 2004). Paterson *et al.* (1979) also detected *R. salmoninarum* in the kidneys of Atlantic salmon returning to rivers in Eastern Canada using an indirect fluorescent-antibody technique. However, the significance of reservoirs of infection in wild and feral salmonid populations in Western Europe is unclear. A PCR-based survey of wild fish from six rivers in England and Wales reported an infection prevalence of 8% in grayling (*Thymallus thymallus*) and 4.8% in Atlantic salmon (Chambers *et al.*, 2008). It has also not been determined whether the original outbreaks of the ‘Dee Disease' in Scottish rivers (Smith, 1964) were caused by introduction of the pathogen from elsewhere (for example, via ova imported from North America), or the represented reoccurrence of a disease that had long been endemic in European populations of Atlantic salmon. In the UK there has also been debate as to the extent to which farmed rainbow trout infected with *R. salmoninarum* pose a risk to farmed (and wild) Atlantic salmon and the best ways to control these potential risks (Murray *et al.*, 2011; Richards, 2011; Murray *et al.*, 2012).

Highly discriminatory *R. salmoninarum* genotyping tools are required to address these questions. Several different molecular typing methods have been developed for *R. salmoninarum* (Grayson *et al.*, 1999, 2000; Rhodes *et al.*, 2000; Alexander *et al.*, 2001). The data generated by these studies point to *R. salmoninarum* being highly clonal with very limited phenotypic and genetic variation. There is, therefore, a need for more powerful methods to resolve sub-lineages within the population and, in doing so, to reconstruct recent evolutionary history and patterns of transmission. To this end, we generated genome-wide single-nucleotide polymorphism (SNP) data using a next-generation sequence platform for 68 strains of *R. salmoninarum* isolated from different host species, and wide temporal and geographical ranges. The data were analyzed using methods pioneered for use with important human pathogens (for example, Harris *et al.*, 2010; Mutreja *et al.*, 2011; Holt *et al.*, 2012). To our knowledge, this study represents the first application of this technology to a strictly animal pathogen.

## Materials and methods

### Bacterial strains and growth conditions

The sample was composed as follows: two of the original isolations from wild Atlantic salmon from the River Dee in 1960, 12 isolates from Norway, 7 isolates from New Brunswick on the East Coast of Canada, 11 isolates from the West Coast of the USA and Canada (including Washington, Oregon and British Columbia), 22 isolates from Scotland, 12 isolates from England and Wales, one single isolate from an eastern brook trout from Alberta, Canada and one isolate from a grayling from Montana, USA. Full details of these isolates are given in Table 1.

Cultures were grown on KDM solid media (Evelyn, 1977) at 15 °C, with colonies isolated and grown for DNA extraction.

### DNA extraction and sequencing

Each participating laboratory separately prepared DNA from the isolates they supplied for the study. In brief, DNA was extracted from freshly grown bacteria harvested directly from solid media, resuspended in 500 μl sterile deionized water, and then centrifuged at 14 000 *g* for 15 min. Or, in other cases, cultures of *R salmoninarum* were grown in KDM broth to an OD_525_ and centrifuged at 10 000 *g* for 20 min. In all cases, the resultant bacterial pellets were then resuspended in 1 ml 10 mM Tris and the DNA extracted as described by Wiens *et al.,* 2002. There were no noticeable differences in the DNA quality in preparations from either solid or broth media (data not shown).

All the isolates (Table 1) were submitted for whole-genome, paired-end sequencing to The Genome Analysis Centre, Norwich, UK. DNA quality and yield was first determined by fluorometry using a Qubit fluorometer (Invitrogen) with QUANT-iT dsDNA assay (Broad Range). The samples were also assessed for RNA contamination using a Qubit fluorometer with QUANT-iT RNA assay (Invitrogen). DNA TruSeq libraries were constructed for each isolate and were run on the Illumina (San Diego, CA, USA) HiSeq 2000 platform in pools of up to 12 libraries per lane.

### DNA TruSeq library construction

The Illumina TruSeq DNA Sample Preparation was used to prepare pooled-indexed paired-end libraries of genomic DNA for subsequent cluster generation (Illumina cBot) and DNA sequencing using the reagents provided in the Illumina TruSeq DNA Sample Preparation v2 Kit. The samples (starting material of ∼1 μg of genomic DNA) were sheared using a sonicator (Covaris (Woburn, MA, USA), S2/LE220) to fragment sizes in the range of 200–700 bp. The ends of the DNA were then repaired and a single A base added to each 3′ end of the DNA fragment to which an indexed adapter binds. A gel size selection method was used (Invitrogen E-Gel, 2% agarose) to select the appropriate sized library that was then enriched by PCR, quantified using an Agilent (Santa Clara, CA, USA) DNA 1000 chip on the Agilent Bioanalyzer 2100, pooled with up to 12 other libraries and sequenced. Library preparation was automated using a Perkin Elmer (Waltham, MA, USA) (formally Caliper) Sciclone NGS Workstation (sonication, size selection, enrichment and pooling are not performed on the Sciclone).

### Data processing, genome alignment and assembly

Close to 1 000 000 000 pairs of reads, each of length 100 bp, were created for the project in total. The raw data have been deposited in the database of the European Bioinformatics Institute, and is available at http://www.ebi.ac.uk. Accession numbers are listed in Table 1. Reads were pre-filtered through the *eliminate_singletons.py*, *eliminate_n_paired.py* and *filter_reads.py* scripts from the BioPython (version 1.60) package (Cock *et al.*, 2009) before assembly, excluding reads that would not properly pair, reads with ambiguous (‘N') base calls and reads with an average PHRED quality score below 20.

We used MAQ v.0.7.1 (Li *et al.*, 2008) to align raw reads to ATCC33209, the reference genome published in GenBank (http://www.ncbi.nlm.nih.gov/genbank. NCBI accession number: NC_010168.1). MAQs *sol2sanger* script was used to transform PHRED scores to the PHRED+33 style. The vast majority of the reads mapped onto the reference genome, providing an average read depth across non-gap regions of 862.0 (Inter-isolates range: 57.2–2012.5). Statistics related to read output, read depth and read mapping is presented in Supplementary File SF3. Reads that mapped equally well to multiple areas of the reference genome, such as reads representing IS994 and ISRs2 sequences, were randomly assigned to one of the possible mapping locations. Variant calls from these areas were considered unreliable and were excluded from further analyses. Non-mapping reads were sorted into a separate file for separate *de novo* assembly in Velvet v.1.2.03 (Zerbino and Birney, 2008), using a k-mer length of 31. However, unmapped reads were all from Bacteriophage phi X 174, which was used as a positive control in the sequencing stage.

To validate these results, we also did *de novo* assembly using a combination of outputs from the DBG assemblers Velvet and ABySS (Simpson *et al.*, 2009), as well as comparative assembly using the AMOScmp-shortreads tool from the AMOS package (Treangen *et al.*, 2011). These assemblies were then merged using the minimus2 tool. This is an abridged version of the pipeline conceived and described by Ji *et al.*, 2011. Comparative evaluation of the output from these two different pipelines was done in Hawkeye (Schatz *et al.*, 2007). MAUVE (Darling *et al.*, 2004) was used to screen for evidence of genomic rearrangements.

### Variant calling and phylogeny reconstruction

SNP calling was done with the default settings in MAQs cns2snp and maq.pl SNPfilter scripts. Furthermore, SNPs with PHRED quality scores of less than 255 were removed from analysis, as did SNPs with a high strand-bias. For a limited number of isolates, MAQ would occasionally produce ambiguous character SNP calls, even though PHRED qualities were consistently high. Closer inspection of the alignment revealed that the issue was caused by a low frequency (<2%) of reads representing an alternative genotype. Although other plausible scenarios could explain this, slight DNA contamination is the most likely. We used the variants represented in the overwhelming majority of reads in these cases. Using SplitsTree4 (Graham *et al.*, 2005), we could find no trace of recombination in our isolates. SNP sequences were then loaded into R (www.r-project.org) and processed using the *ape* (Paradis *et al.*, 2004) and *phangorn* (Schliep, 2011) packages, in conjunction with the maximum-likelihood estimator PhyML (Guindon *et al.*, 2010), to infer the optimal phylogenetic substitution model for the data. Several models were suggested as an outcome of this analysis, but the generalized time-reversible model (Tavaré, 1986) without rate variation among sites obtained the lowest AIC score (Akaike, 1974), and was therefore chosen. Trees for both the full SNP alignment and pseudogene SNPs only were created with the MrBayes (Ronquist and Huelsenbeck, 2003) plugin in Geneious (version 6.0.3), using a generalized time-reversible-substitution model with a uniform site distribution. The Markov Chain Monte Carlo settings were set to include 10 000 000 generations with subsampling done every 2000th step. Burn-in was set to 1 000 000 generations, and the unconstrained branch length option was used. The tree was subsequently annotated in FigTree (http://www.tree.bio.ed.ac.uk/software/figtree/).

### Bayesian Markov Chain Monte Carlo analyses

A dated phylogeny was constructed by means of BEAST (Drummond and Rambaut, 2007; Drummond *et al.*, 2012), based on the multiple alignment of all the SNPs that were not located in paralogous genes, using an uncorrelated lognormal relaxed clock with the generalized time-reversible model. Date of collection was used to estimate the divergence times of isolates. Two analyses were run for 600 000 000 generations, sampling every 30 000 generations. We combined the results from the two independent runs through LogCombiner (excluding the first 10% generations from each analysis). TRACER was used to evaluate the convergence of the combined analysis, the first 10% generations were discarded as burn-in; we corroborated that the effective sample size of all the parameters were greater than 200, and ensured that the trace plots of the likelihood scores randomly oscillated within a stable range. The mean rate of the molecular clock for the whole genome was calculated by multiplying the mean rate of the uncorrelated lognormal clock for the SNP collection by the SNP density in the genome.

### Ancestral character state reconstruction

Mesquite (Maddison and Maddison, 2011) was used to reconstruct nodal character states based on terminal taxa values. Phylogenetic trees were constructed from pseudogene SNPs only. Both a parsimonious and a maximum-likelihood approach were used. For both approaches, geographical data were simplified to represent the country of origin in an unordered, character matrix. In the likelihood estimate, the one-parameter Markov k-state probability model (Lewis, 2001) was used.

### Accession numbers

The sequence data for all the isolates were deposited in the European Bioinformatics Institute Short Read Archive under the accession numbers ERR327904 to ERR327971inclusive.

## Results

### Phylogenetic analysis of *R. salmoninarum*

**Figure 1:**
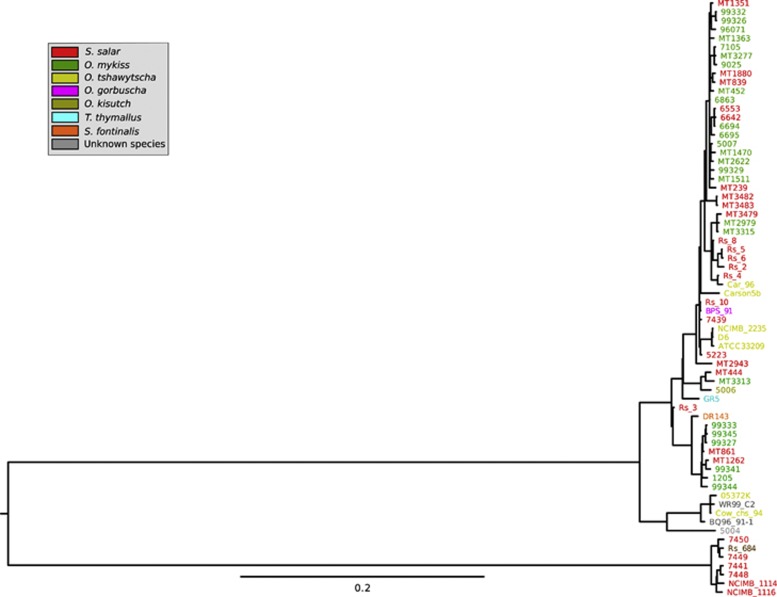
Phylogenetic tree of the 68 isolates of *R. salmoninarum* included in this study, showing all lineages. The evolutionary history was inferred using a Bayesian Markov Chain Monte Carlo approach, with a generalized time-reversible model (Tavaré, 1986), through the MrBayes (Ronquist and Huelsenbeck, 2003) plugin in Geneious. The consensus tree is taken to represent the evolutionary history of the taxa analyzed. The tree is drawn to scale, with branch lengths measured in the number of substitutions per site. All ambiguous positions were removed for each sequence pair. There were a total of 3600 positions in the final data set. The leftmost node represents a hypothetical most recent common ancestor. The above and bottom branches from this node represent lineages 1 and 2, respectively. Isolates are color coded according to the host: green, rainbow trout; red, Atlantic salmon; yellow, Chinook salmon; pink, pink salmon; teal, Grayling; gold, Coho salmon; orange, Eastern brook trout; brown, brown trout; gray, not known.

**Figure 2:**
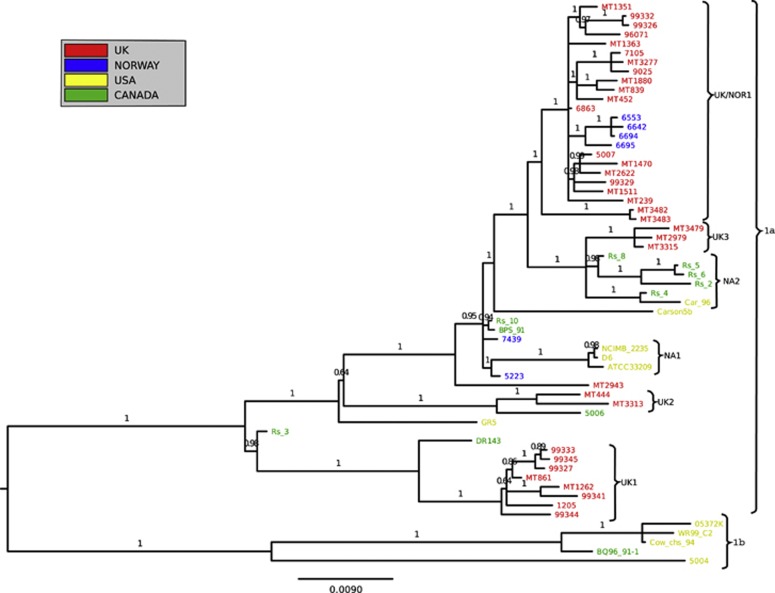
Detail of lineage 1. The phylogenetic tree was constructed as described under Figure 1. Posterior probability values are shown on each branch. For the detailed look at subgroup UK/NOR1, branches have been transformed so as to no longer represent evolutionary distance. Isolates are color coded according to their geographical origin: red, UK; blue, Norway; green, Canada; yellow, USA.

A total of 3600 high-quality core-genome SNPs were identified (see Supplementary File 1), corresponding to one SNP every 876 bases. These were evenly distributed across the genome. There was no evidence of genomic rearrangement. Phylogenetic analysis resolved two major sub-lineages; lineage 1 and lineage 2 (Figure 1). Lineage 1 encompassed 90% of the studied isolates (61/68). These isolates were recovered from seven of the eight different host species, from the full range of geographical locations, and over a 50-year period (1960–2009). Despite this geographical, temporal and ecological diversity, lineage 1 isolates exhibit very low levels of genetic diversity. The average pairwise nucleotide diversity (π) across the whole genome (3.15 Mb) within lineage 1 is 0.00005, corresponding to an average of 167 SNP differences between pairs of strains. This paucity of variation is indicative of a slow rate of evolution, recent common ancestry of the population with global dissemination or both. The seven isolates of lineage 2 (including the isolates recovered from the River Dee in 1960), were all isolated from the UK and Norway and are exclusively associated with the genus *Salmo*; six from *Salmo salar* (Atlantic salmon) and one from *Salmo trutta* (brown trout). The average number of SNP differences between lineage 2 isolates is 80, approximately half that of the lineage 1 isolates (π=0.000025). The level of diversity between lineages is ∼20-fold greater than the diversity within them, with an average inter-lineage difference of 2431 SNPs (π=0.0008). The majority of the clades in the phylogeny are supported by high (>0.9) PPS (posterior probability scores) (See Figure 2; all clades from linage 2 have complete support as evidenced by PPS of 1), indicating the phylogenetic analysis has not been significantly confounded by recombination. To confirm this, we checked for the presence of recombination using the Phi test and splits decomposition as implemented in SplitsTree 4 (Graham *et al.*, 2005). We carried out this analysis on the two major lineages separately, as the relatively large distance between them would impair visual inspection of the network. The Phi test did not find significant evidence for recombination either for Lineage 1 (*P*=0.86) or for Lineage 2 (*P*=0.1). Although inspection of the splits decomposition networks reveals very little reticulation for Lineage 1, slight reticulation is suggested between the seven strains of Lineage 2 (Supplementary Figure S1). The evidence thus points to a near absence of recombination, although a very limited degree of recombination cannot be excluded in Lineage 2.

### Inferring patterns of global transmission

Lineage 1 contains a mixture of North American and European isolates, whilst lineage 2 is restricted to Europe. We used BEAST (Drummond and Rambaut, 2007; Drummond *et al.*, 2012) for evolutionary analysis using an uncorrelated relaxed, lognormal clock. The mean clock rate was estimated as 3.324 × 10^−4^ mutations/site/year for our 3600 SNPs, corresponding to an overall rate of 3.8 × 10^−7^ mutations/genome/year. This figure represents the mean rate and may not be representative of individual lineages. The time to most recent common ancestor of all samples was dated to ∼1239 years ago (95% CI 444–2720 years ago) (Supplementary Figure S2) However, it should be noted that the credible interval is rather wide leading to a not very precise point estimate; hence, caution should be exercised in considering this estimate. The lineages thus started to diverge at some point in time beyond this estimate. Lineage 1 can be further subdivided into lineage 1a and lineage 1b (Figure 2). Lineage 1b corresponds to five isolates from North America, consistent with a North American origin for this group. In contrast, Lineage 1a consists of isolates recovered from both North America and Europe. We note a general trend from the Lineage 1 tree that the most basal isolates tend to be of North American origin, supporting the view that this lineage emerged in North America and has more recently been transmitted to Europe rather than the other way around. For example, isolate Rs_3 was recovered from an Atlantic salmon in New Brunswick, Canada, and isolate DR143, which was isolated from a brook trout (*Salvelinus fontinalis*) from Alberta, Canada, both positioned in group 1a, basally to a cluster of eight isolates from the UK (labeled UK1 on Figure 2). The UK1 cluster may therefore represent a transmission event from Canada to the UK and, according to the BEAST analysis, this is most likely to have occurred ∼66 years ago (95% CI 40–120 years ago). The eight isolates corresponding to the UK1 cluster originate from fish farms in Wales, Scotland and England, indicating dissemination throughout UK farms subsequent to this transmission event that affected at least two commercial host species: Atlantic salmon (MT1262 and MT861) and rainbow trout (99 333, 99 327, 99 341, 99 344, 1205 and 99 345). The large cluster of 22 isolates from the UK and Norway (UK/NOR1) is also consistent with a single recent introduction ∼43 years ago (95% CI 30–65 years ago.), followed by rapid spread between the UK and Norway, affecting multiple host species. Finally, two small clusters of UK isolates are evident in Lineage 1a (UK2, UK3), which may also represent independent introductions and transmission across multiple host species within the UK.

**Figure 3:**
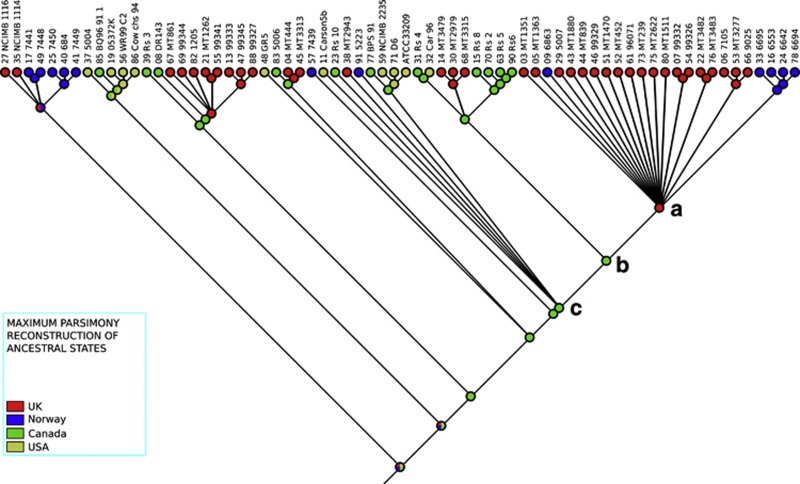
Ancestral state reconstruction of the geographical origin. Nodes in the trees have been estimated from a maximum parsimony evaluation of terminal values, where country of origin has been evaluated in an unordered, categorical matrix. The tree is not drawn to scale. The legend is as follows: red, UK; blue, Norway; green, Canada; yellow, USA. Nodes A, B and C are referenced in the text. The tree was calculated by using SNPs from open reading frames (ORFs) annotated as pseudogenes in the ATCC33209 genome.

The inferences above are based largely on the casual inspection of the tree. In order to investigate the origins and transmission history of the *R. salmoninarum* isolates within a more robust framework, we performed ancestral state reconstruction using Mesquite (Maddison and Maddison, 2011). We used both parsimony (Figure 3) and maximum-likelihood (Supplementary Figure S3)-based methods. In both cases, geographic origin was treated as a categorical variable with a uniform cost of switching. This parsimony analysis strongly supports the inferences discussed above in predicting a likely North American origin for the UK1, UK2, NA1, UK3 and NA2 groups. Although the immediate ancestor of the UK/NOR1 is predicted to originate from the UK (Figure 3; node A), the analysis points to a character switch before the emergence of this clade as the most parsimonious states at nodes B and C are of Canadian origin. This character switch thus reflects a transmission from North America to the UK.

We also considered proportional likelihoods of each state at the different internal nodes in the tree as given in Supplementary File SF2. This maximum-likelihood approach unsurprisingly confirms that the immediate ancestor of the UK/NOR1 group (node A in Figure 3) was very likely of UK origin (node 52 in SF2; proportional likelihood score for UK origin=1.0). Moving one node back in the tree (node B in Figure 3) the proportional likelihoods of a UK and North American origin were found to be approximately equal (node 76 in SF2; proportional likelihood=0.44 for UK and 0.48 for Canada). However, moving back one node (node C in Figure 3) the maximum-likelihood analysis provides far stronger support for a North American origin, and very weak evidence for a UK origin (node 77 in SF2; proportional likelihood=0.05 for UK and 0.75 for Canada). This analysis therefore points to a switch (transmission) from North America to UK either between node C and node B (as suggested by maximum likelihood), or between node B and node A (as suggested by maximum parsimony) (Figure 3).

### Detecting outbreaks

In addition to providing evidence concerning large-scale patterns of transmission, next-generation sequence data can also distinguish between samples of contemporaneous isolates recovered from a local setting. The technology allows the analyst to accurately assess whether or not concurrent presentations of disease form an outbreak or not. For instance, UK/NOR1 cluster isolates 6642, 6694 and 6553, recovered as part of disease outbreak investigations in the region of Sør-Trøndelag in 2008, were very closely related, differing at only five polymorphic sites. Strikingly, these three isolates also demonstrate free transmission between hosts; isolates 6642 and 6553 are from the same *S. salar* farm in Hemne, whereas 6694 and 6695 are from a nearby *O. mykiss* farm. In other cases the technology demonstrated that contemporaneous strains isolated from a single farm are not always epidemiologically linked, but may in some cases correspond to a mixture of the major lineages circulating throughout the UK and beyond. For example, isolates 99329, 99333 and 99345 were all isolated in 1998 from the same site in Wales but the latter two isolates belong to the cluster UK1, whilst 99323 belong to UK/NOR1.

## Discussion

The very low density of polymorphisms and almost total conservation of gene content confirm that *R. salmoninarum* is a highly clonal pathogen. A very low level of genome diversity has been noted in other highly specialized intracellular bacterial pathogens, notably *Mycobacterium tuberculosis*, which is phylogenetically related to *R. salmoninarum* and also causes a chronic granulomatous disease. Our estimate for a mean clock rate of 3.8 × 10^−7^ mutations/bp/year is higher than a recent estimate for *M. tuberculosis* of 7.3 × 10^−8^ (Ford *et al.*, 2011. Scaled up from mutations/bp/day), and notably much higher than rates for *Yersinia pestis* that has been estimated at 8.6 × 10^−9^ (Morelli *et al.*, 2010). However, *R. salmoninarum* appears to mutate much more slowly than other major human pathogens such as *Staphylococcus aureus*, for which the rate was recently reported as 1.3 × 10^−6^ (Holden *et al.*, 2013).

Although isolates were recovered from many different species—Atlantic salmon, grayling, brook trout, rainbow trout and other *Oncorhynchus spp*.—they are almost indistinguishable genetically, indicating free interspecies transmission and broad virulence properties. Our data therefore suggest that previously observed differences in host susceptibility to BKD (Starliper *et al.*, 1997) are likely to reflect host and/or environmental factors rather than variation within the pathogen. The limited genetic diversity of UK isolates in particular may be related to the controls implemented in response to the 1937 Diseases of Fish Act, which prohibited import of live salmonids into the UK and also made it illegal to import salmonid ova and any live freshwater fish species without a license (Hill, 1996). This has likely limited associated importation of pathogens such as *R. salmoninarum,* which could be one of the reasons that a relatively limited range of subtypes of this, and another important bacterial pathogen, *Yersinia ruckeri* (Davies, 1991; Wheeler *et al.,* 2009) appear to be present within UK farmed fish. It has previously been noted that the main subtype of *Y. ruckeri* circulating in farmed UK rainbow trout also shows limited diversity and is different from the main strains circulating in Europe (Davies, 1991; Wheeler *et al.*, 2009), suggesting these controls have restricted pathogen exchange between the UK and mainland Europe aquaculture production systems.

The data reveal evidence concerning long-term geographical structuring and global transmission. The construction of a high-resolution SNP-based phylogeny robustly resolved two major lineages of *R. salmoninarum*, lineage 1 and lineage 2. Given the extremely low divergence within lineage 1, it can be speculated that its rapid geographical and ecological dispersal, through different oceans and species of salmonids, has likely been facilitated by anthropogenic means. Commercial activities over the last 150 years have provided ample opportunities for such a spread to occur. Live salmonids and ova have been traded on a global scale for aquaculture, recreational angling and fishery stock enhancement purposes since the mid-19th century (Halverson, 2010). The last 40 years in particular have witnessed increasingly intensive and vertically integrated production, initially of rainbow trout and latterly of Atlantic salmon. Eggs are typically produced in dedicated broodstock facilities before being moved to hatcheries for production of fry, which are then moved again to ongrowing sites (either seawater cages for Atlantic salmon, or larger tanks or raceways for rainbow trout). As with other intensive livestock production systems, frequent transport means that breaches in biosecurity can lead to the transmission of pathogens between premises (Plarre *et al.*, 2012). Furthermore, evidence exists that other highly clonal bacterial diseases of salmonids have spread between continents in similar ways, likely via egg and live fish movements. (Davies, 1991; Garcia *et al.*, 2000; Wheeler *et al.*, 2009).

In contrast to the global distribution of lineage 1, the absence of North American isolates in lineage 2 suggests that this lineage may represent a long-term endemic disease in European waters. Lineage 2 isolates all came from the North Sea area (including tributaries). The early outbreaks in Norway in the 1980s, all of lineage 2 origins, were reportedly in re-stocking hatcheries based on the capture and stripping of wild Atlantic salmon brood stock, and thus may have been caused by the pathogen present in wild populations. It is notable that the implementation of strict biosecurity measures and screening efforts in the late 1980s coincided with a significant reduction in outbreaks of BKD in Norway (Wiens and Dale, 2009; Johansen *et al.*, 2011). The origin of lineage 1 is more unclear. The estimated phylogenetic division between the two main lineages (∼1000 years ago) clearly pre-dates commercial activity. It is possible that lineage 1 emerged independently within a geographically or ecologically isolated population of Atlantic salmonid before being transferred into Pacific salmonid populations. In support of this, BKD was not reported as a problem in Pacific salmon hatcheries on the West Coast of the US (Earp *et al.*, 1953) until much later than East Coast hatcheries (Belding and Merrill, 1935). This is also consistent with the suggestion that the pathogen has co-evolved for longer with Atlantic salmonids (*Salmo* and *Salvelinus*), thus potentially explaining why Pacific salmon species are reportedly more susceptible to this pathogen (Evenden *et al.*, 1993; Starliper *et al.*, 1997). The relatively limited recovery of lineage 2 isolates as opposed to lineage 1 isolates in the UK and Norway was noteworthy. Further research is needed to establish whether any genetic advantage in either of the lineages can explain this asymmetry in distribution.

In general, the accuracy of determination of the relatedness of isolates demonstrated here was not possible with previously employed typing methods (Grayson *et al.,* 1999,^2000^,Rhodes *et al.,* 2000). As an example, previous random amplified polymorphic DNA-based analysis (Grayson *et al.*, 2000) grouped the DR143 and Cow_chs_94 isolates on the same terminal branch, whereas our dated phylogenetic trees reveal that the most recent common ancestor of these isolates existed about 360 years ago. (95% CI ∼150–700 years ago). This clearly illustrates the superiority of the next-generation sequence technology for outbreak determination problems.

Our data and analysis have delineated two major European clusters within Lineage 1a (UK1 and UK/NOR1) that emerged <70 years ago, probably as a result of transmission from North America, and have spread between fish farms in the UK and Norway infecting both rainbow trout and Atlantic salmon. Support for this comes from the observation that the North American lineage 1 isolates tend to be more diverse (and basal) than the European lineage 1 isolates, suggesting the former reflect the ‘ancestral' population and the latter consist of independent introductions (bottlenecks). Our reconstruction of ancestral states analysis also points to the North American origin of most sub-lineages in Lineage 1. In summary, this points to transatlantic commercial activity as a likely factor in the European emergence and spread of lineage 1. However, it should be emphasized that all this refers only to the major European lineage 1 clades that we have sampled, and it is not possible to extrapolate from this to general conclusions about rates of transmission in either direction, and significant transmission from Europe to North America through commercial activity may also have taken place.

## Conclusion

The application of whole-genome SNP-based comparisons has offered a range of insights into the likely microevolutionary relationships of this important fish pathogen that hitherto would not have been possible. The analysis reveals an unexpected deep phylogenetic division in the population, hinting at historical allopatry, evidence for transatlantic transmission and spread over the scale of decades and even proof of principle that the approach can be used to identify single outbreak strains on a very local scale. It is recommended that this methodology should also be applied for studies of other veterinary pathogens and environmental microorganisms, particularly those with very limited genetic intra-species variation.

## Figures and Tables

**Table 1:** *R. salmoninarum* isolates used in the study

*Isolate*	*Geographic origin*	*Year*	*Source*^a^	*Alternative ID*	*GenBank/ EBI accession number*
Rs 10	New Brunswick, Canada	2009	*Salmo salar* (sw)		ERR327945
Rs 2	New Brunswick, Canada	2005	*S. salar* (sw)		ERR327951
Rs 3	New Brunswick, Canada	2005	*S. salar* (fw)		ERR327947
Rs 4	New Brunswick, Canada	2006	*S. salar* (sw)		ERR327946
Rs 5	New Brunswick, Canada	2007	*S. salar* (sw)		ERR327950
Rs 6	New Brunswick, Canada	2007	*S. salar* (sw)		ERR327953
Rs 8	New Brunswick, Canada	2008	*S. salar* (sw)		ERR327944
BPS 91	Nanaimo, BC, Canada	1991	*Oncorhynchus gorbuscha*		ERR327952
BQ96 91-1	Nanaimo, BC, Canada	1996	*Oncorhynchus kisutch*		ERR327963
DR143	Alberta, Canada	1972	*Salvelinus fontinalis (*fw)^b^		ERR327954
5006	Bella Bella, BC, Canada	1996	*O. kisutch* (sw)	960046	ERR327942
5223	Kvinnherad, Hordaland, Norway	2005	*S. salar* (sw)	2005-50-579	ERR327964
6553	Hemne, Sør-Trøndelag, Norway	2008	*S. salar* (sw)	2008-09-495	ERR327955
6642	Hemne, Sør-Trøndelag, Norway	2008	*S. salar*	2008-06-633	ERR327956
6694	Hemne, Sør-Trøndelag, Norway	2008	*Oncorhynchus mykiss* (sw)		ERR327962
6695	Hemne, Sør-Trøndelag, Norway	2008	*O. mykiss* (sw)	2008-06-631	ERR327968
6863	Osterøy, Hordaland, Norway	2009	*O. mykiss* (sw)		ERR327965
7439	Sognefjorden, Sogn og Fjordane, Norway	1984	*S. salar*	1984-40.992	ERR327971
7441	Storfjord, Møre og Romsdal, Norway	1985	*S. salar*	1985-09-667	ERR327966
7448	Stranda, Møre og Romsdal, Norway	1986	*S. salar*	1986-09-4366	ERR327970
7449	Skjervøy, Troms, Norway	1987	*S. salar*	1987-09-932	ERR327969
7450	Askøy, Hordaland, Norway	1987	*S. salar*	1987-09-1185	ERR327967
684	Aurland, Sognefjorden, Norway	1987	*S. trutta* (fw)		ERR327958
1205	UK	2001	*O. mykiss*	3104-67	ERR327930
5007	Scotland	2005	*O. mykiss*	0180-18	ERR327923
7105	UK	2007	*O. mykiss* (fw)	P0416 T83 10-3 2	ERR327932
9025	Yorkshire, England, UK	2009	*O. mykiss* (fw)	16251-1	ERR327912
96071	England, Hampshire, site Z, UK	1996	*O. mykiss* (fw)		ERR327927
99326	Wales, site Y, UK	1999	*O. mykiss* (fw)	2119-8	ERR327938
99327	UK	1997	*O. mykiss* (fw)	970313-2	ERR327931
99329	Wales, site X, UK	1998	*O. mykiss* (fw)	980036-125	ERR327937
99332	Wales, site Y, UK	1999	*O. mykiss* (fw)	2119-3	ERR327943
99333	Wales, site X, UK	1998	*O. mykiss* (fw)	980036-102	ERR327921
99341	Hampshire, site Z, England, UK	1998	*O. mykiss* (fw)	980109-20	ERR327949
99344	Hampshire, England, UK	1998	*O. mykiss* (fw)	980106-1.1.5	ERR327940
99345	Wales, site X	1998	*O. mykiss* (fw)	980070-18	ERR327948
NCIMB 1114	River Dee, Scotland, UK	1962	*S. salar* (fw)^b^	5005	ERR327908
NCIMB 1116	River Dee, Scotland, UK	1962	*S. salar* (fw)^b^	96056	ERR327907
MT239	Scotland, UK	1988	*S. salar*		ERR327913
MT1363	Strathclyde, Scotland, UK	1993	*O. mykiss* (sw)		ERR327920
MT3277	Dumfries and Galloway Site A, Scotland,UK	2008	*O. mykiss* (fw)		ERR327926
MT3313	Central, Scotland, UK	2008	*O. mykiss* (fw)		ERR327925
MT3315	Strathclyde Site B, Scotland, UK	2008	*O. mykiss* (fw)		ERR327928
MT1262	Highlands, Scotland, UK	1992	*S. salar* (fw)		ERR327922
MT1351	Highlands, Scotland, UK	1993	*S. salar* (sw)		ERR327904
MT1470	Tayside, Scotland, UK	1994	*O. mykiss* (fw)		ERR327910
MT1511	Strathclyde Site B, Scotland, UK	1994	*O. mykiss* (fw)		ERR327914
MT1880	Strathclyde, Scotland, UK	1996	*S. salar* (sw)		ERR327909
MT2622	Strathclyde, Scotland, UK	2002	*O. mykiss* (sw)		ERR327929
MT2943	Highlands, Scotland, UK	2005	*S. salar* (sw)		ERR327936
MT2979	Highlands, Scotland, UK	2005	*O. mykiss* (fw)		ERR327935
MT3106	Strathclyde, Scotland, UK	2006	*O. mykiss* (fw)		ERR327939
MT3479	Orkney, Scotland, UK	2008	*S. salar* (sw)		ERR327933
MT3482	Strathclyde, Scotland, UK	2009	*S. salar* (sw)		ERR327934
MT3483	Strathclyde, Scotland, UK	2009	*S. salar* (sw)		ERR327941
MT444	Western Isles, Scotland, UK	1988	*S. salar* (sw)		ERR327916
MT452	Dumfries and Galloway Site A, Scotland,UK	1988	*O. mykiss* (fw)		ERR327918
MT839	Highlands, Scotland, UK	1990	*S. salar* (sw)		ERR327917
MT861	Scotland	1990	*S. salar* (sw)		ERR327919
Car 96	Washington State, USA	1996	*O. tshawytscha*		ERR327957
D6	Oregon, USA	1982	*O. tshawytscha*		ERR327961
GR5	Montana, USA	1997	*T. thymallus* (fw)^b^	980036-87	ERR327959
WR99 c2	Washington State, USA	1999	*O. kisutch*		ERR327960
NCIMB 2235	Oregon, USA	1974	*O. tshawytscha* (sw)	ATCC33209	ERR327911
05372K	Grande Ronde Basin, Oregon, USA	2005	*O. tshawytscha* (sw)		ERR327906
Carson 5b	Confluence Tyee Creek & Wind River, WA, USA	1994	*O. tshawytscha* (fw)		ERR327905
Cow-chs-94	Cowlitz River, Washington	1994	*O. tshawytscha* (fw)		ERR327915
ATCC 33209^c^	Oregon, USA	1974	*O. tshawytscha* (sw)		NC_010168.1
NCIMB 1111^d^	—	Not known	Not known	5004	ERR327924

Abbreviations: fw, fresh water; sw, sea water.

The complete history for some of the isolates is not known.

a Isolates recovered from fw or sw, where known.

b Isolate recovered from a wild fish species, all other isolates were recovered from farmed fish or not known.

c Used as a reference in this study. Sequence data downloaded from Genbank.

d NCIMB 1111 was deposited in the NCIMB culture collection in Aberdeen after 1960 by I Smith, who also deposited isolates NCIMB 1114 and NCIMB 1116 recorded as being isolated by her from wild *S. salar* from the River Dee in the early 1960s. NCIMB 1111 was reportedly isolated by Ken Wolf. Ken Wolf was a highly active US fish disease researcher who worked on US and Canadian strains of the pathogen in the 1950s and 1960s. It thus appears most likely that this isolate was a North American strain provided by Ken Wolf to I Smith to assist her with her studies on Dee disease (although this cannot be proven).
