# Supplementary figures and images for: Microevolution of Renibacterium salmoninarum: evidence for intercontinental dissemination associated with fish movements
Source: ISME J. 2013 Oct 31;8(4):746–56. doi: 10.1038/ismej.2013.186 (PMC3960531; doi:10.1038/ismej.2013.186)

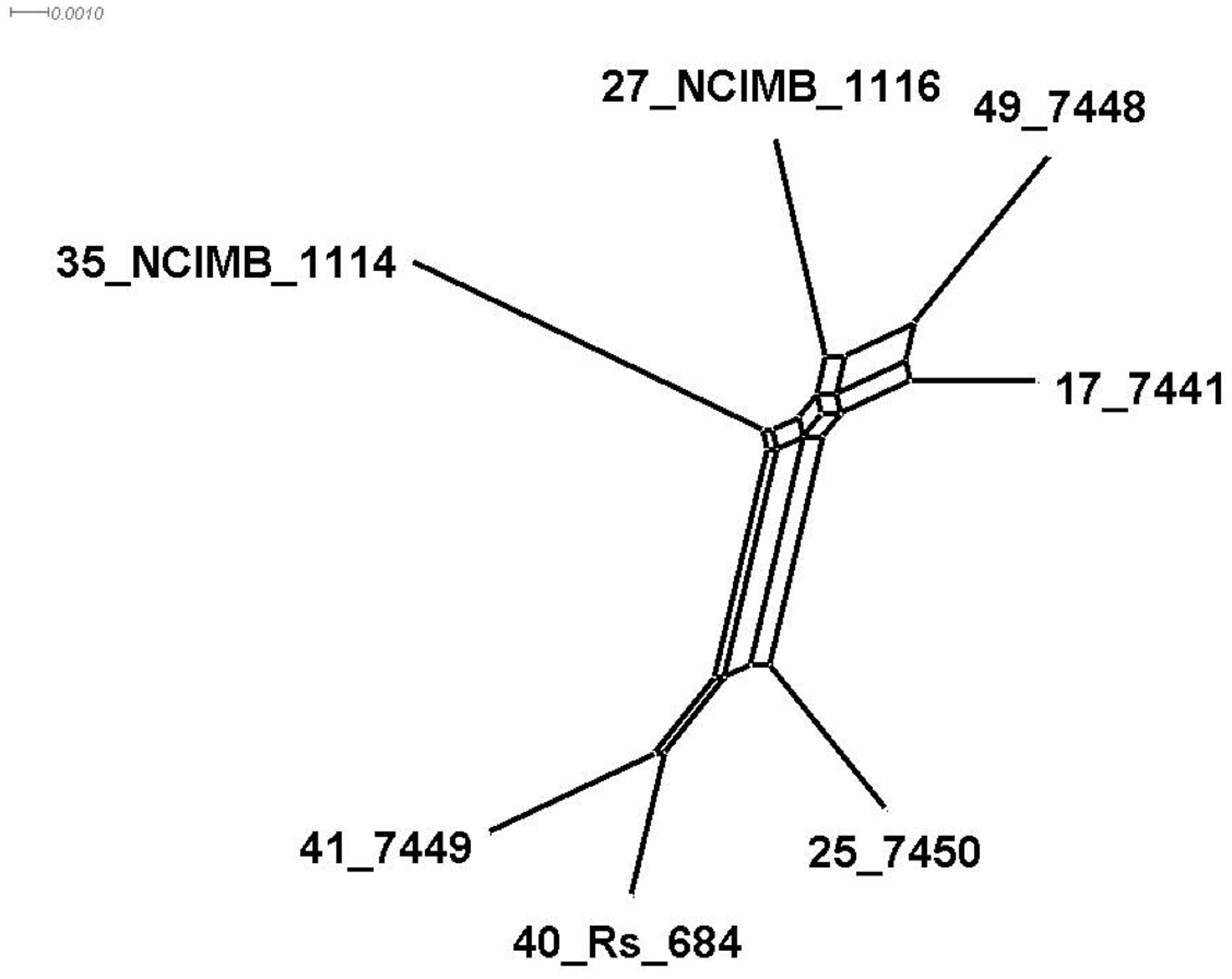

Supplement: Supplementary Figure 1 [file ismej2013186x1.tif]

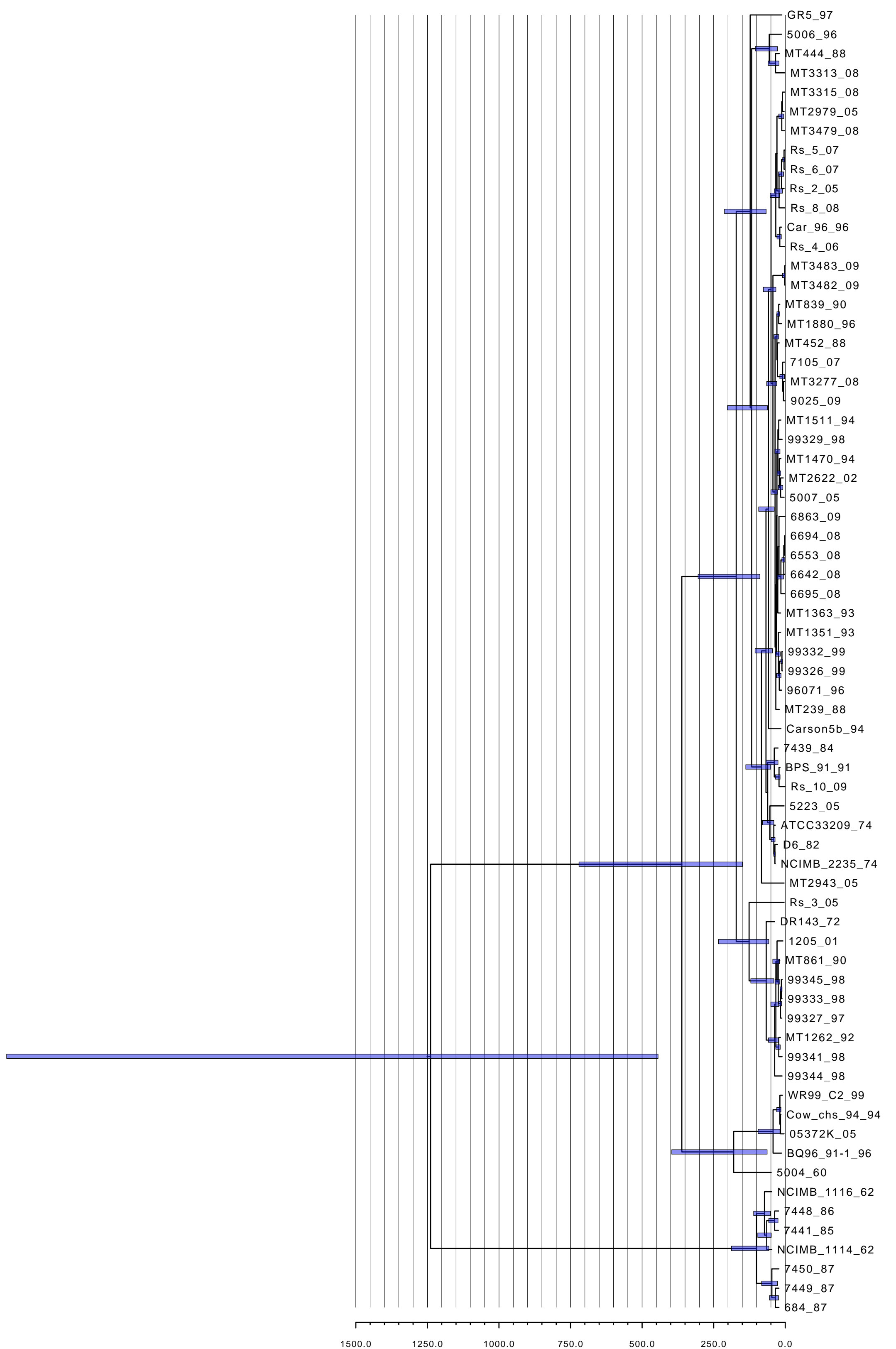

Supplement: Supplementary Figure 2 [file ismej2013186x2.pdf]

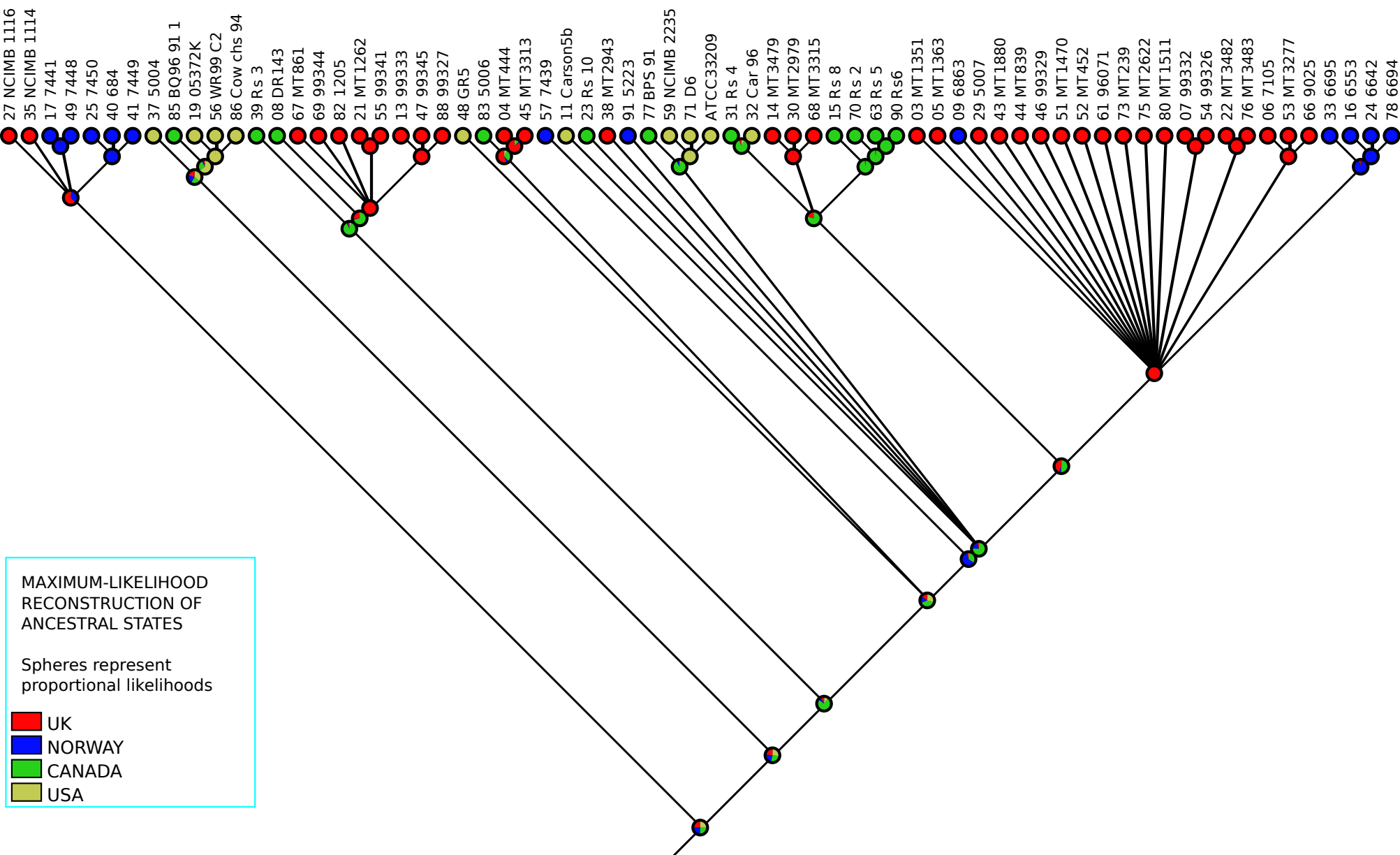

Supplement: Supplementary Figure 3 [file ismej2013186x3.pdf]
