# Supplementary material for: Microevolution of Renibacterium salmoninarum: evidence for intercontinental dissemination associated with fish movements
Source: ISME J. 2013 Oct 31;8(4):746–56. doi: 10.1038/ismej.2013.186 (PMC3960531; doi:10.1038/ismej.2013.186)
Supplement: Supplementary Legends [file ismej2013186x7.doc]

Supplementary figure 1 (S1) Recombination analysis of lineage 2, showing slight reticulation.

Supplementary figure 2 (S2): Dated Bayesian phylogeny constructed via BEAST. An uncorrelated lognormal relaxed clock model was used to construct the tree based on the concatenated alignment of all the SNPs. The horizontal bars show the 95% HPD intervals for the divergence time estimates.

Supplementary figure 3 (S3) Ancestral state reconstruction of node values in the pseudogene-SNP-based tree, using a maximum-likelihood approach under the one-parameter Markov k-state model. Nodes are drawn to represent the proportional likelihoods.

Supplementary file 1 (SF1): Complete database of the 3600 SNPs identified in this study. SNPs and their positions relate to the forward strand of the ATCC33209 genome. I = intergenic mutation; M = missense (nonsynonymous) mutation; N = nonsense (stop) mutation; S = synonymous mutation.

Supplementary file 2 (SF2): Proportional likelihood values of the tree in Figure S4.

Supplementary file 3 (SF3): Statistics related to read output, average read depth and percentage of total reads mapped to the reference genome for each individual isolate.
